# The Determinants of the Quality of Life of Gastroesophageal Reflux Disease Patients Attending King Saud University Medical City

**DOI:** 10.7759/cureus.9505

**Published:** 2020-08-01

**Authors:** Sulaiman A Alshammari, Abdulaziz M Alabdulkareem, Khaled M Aloqeely, Muath I Alhumud, Saud A Alghufaily, Yazeed I Al-Dossare, Naser O Alrashdan

**Affiliations:** 1 Family and Community Medicine, King Saud University, Riyadh, SAU

**Keywords:** gastroesophageal reflux disease, gerd, quality of life, qol, gerd-hrql, saudi arabia

## Abstract

Background

Gastroesophageal reflux disease (GERD) is a common gastrointestinal (GI) tract disease and has an adverse effect on the quality of life (QoL) of patients. Studies on the QoL of GERD patients would increase awareness among healthcare providers about the magnitude of the problem and how to manage it.

Objectives

This study aimed to determine the essential variables that affected the QoL of GERD patients at King Saud University Medical City (KSUMC), Riyadh, Saudi Arabia.

Method

This was a cross-sectional study performed using quantitative questionnaires, which was conducted at KSUMC during the period from September 1, 2019, to April 1, 2020. We used the gastroesophageal reflux disease health-related quality of life (GERD-HRQL) questionnaire. Additionally, the authors collected information about sociodemographic variables and concomitant diseases from each participant.

Results

The study included 200 participants; 58.5% of them were males. Around 34.9% of participants in the age group A (18-34 years) had poor QoL compared to 57.5% and 50% of participants from the counterpart groups, respectively. There was a statistically significant difference in the QoL among various age groups (p: 0.006). Furthermore, 58.9% of obese participants had poor QoL; there was a statistically significant difference in the QoL between normal-weight and obese groups (p: 0.013).

Conclusion

The QoL of GERD patients was found to be affected negatively by increasing age and high body mass index (BMI). None of the other demographic variables and concomitant diseases had any significant effect on the QoL of the participants.

## Introduction

Gastroesophageal reflux disease (GERD) is a common gastrointestinal (GI) tract disease, and it is the most common GI-related diagnosis given during in-office visits [1]. The estimated prevalence of GERD in Saudi Arabia is quite variable, ranging from 15-45.4%, with a large study reporting a rate of 28.7%, which is quite high [2-4] and exceeds the prevalence in eastern Asia (lower than 10%), western Asia (10-20%), and the estimated worldwide prevalence (15-25%) [5-7]. GERD is a disorder that develops when the gastric reflux causes troubling symptoms and complications [8]. In terms of presentation, GERD is divided into esophageal and extra-esophageal syndromes [8]. Patients generally report a burning feeling in the retrosternal area, rising into the chest and radiating toward the neck, throat, and, occasionally, the back [8]. Nighttime heartburn may cause sleeping difficulties and impair next-day function. Nocturnal awakenings are prevalent among GERD-affected individuals, and chronicity is strongly predicted by insomnia disorder, sleep dissatisfaction, difficulty in initiating sleep, major depressive disorder, and various medical disorders [9]. Individuals who report frequent GERD symptoms commonly experience nocturnal symptoms [10]. GERD, apart from its serious effect on the quality of life (QoL), can progress to many complications such as esophageal cancer [11,12]. It has been reported that all aspects and domains of the QoL are impaired by GERD [13]. Risk factors associated with the development of GERD include age, obesity, lifestyle factors such as smoking, lack of physical activity, and nutrition. Additionally, genetic factors may play an adjuvant role [14-16]. The QoL is defined as "the general well-being of individuals and societies, outlining negative and positive features of life" [17].

People who suffer from frequent GERD symptoms tend to have significantly impaired health-related quality of life (HRQL). Furthermore, nocturnal GERD symptoms exacerbate HRQL impairment. HRQL refers to people's capacity to perform daily activities (i.e., functioning) in addition to their perspective on life (i.e., well-being) and subjective management of their health condition [18]. Studies related to QoL in Saudi Arabia are scarce [19]. Achieving a vibrant society is one of the three main pillars of Saudi Vision 2030, which has put the fulfillment of a healthy life as a high-level objective, indicating its importance [20]. The subject of QoL has been accorded a complete program of its own in Saudi Vision 2030; this program is ambitiously aiming at making Saudi Arabia a top living destination for both Saudi citizens and other residents, by focusing on improving the individual's lifestyle and enhancing the QoL [21]. Studies on the QOL of GERD patients would increase awareness among healthcare providers about the magnitude of the problem and how to manage it. The objective of this study was to determine the essential variables that affected the QoL of GERD patients at King Saud University Medical City (KSUMC).

## Materials and methods

The authors conducted a cross-sectional study using self-administered quantitative questionnaires. The study population included patients attending KSUMC in Riyadh, Saudi Arabia, from September 1, 2019, to April 1, 2020. We proceeded based on the assumption that 7.8% of GERD patients reported symptoms that affected their daily life activity [19]. With a precision of within 4% and at Za/2=1.96, 173 participants constituted the sample size of this study. We added 20% to account for non-response. So, the final study sample size was 208 participants. We used a non-probability consecutive method as a sampling technique with the following inclusion criteria: Saudi nationals who were 18 years or older, diagnosed with GERD, who spoke Arabic, and agreed to participate in the study.

To assess GERD's impact on the patients' QoL, we used the gastroesophageal reflux disease health-related quality of life (GERD-HRQL) questionnaire developed by Vic Velanovich (USA). This questionnaire assesses the severity of the symptoms of GERD, by focusing on its typical symptoms. It is self-explanatory and composed of 10 items that examine the frequency of heartburn, regurgitation, difficulty in swallowing, bloating, and the burden of GERD medications in the past two weeks. Each item is scored from zero to five with a maximum rating of 50, with a higher rating indicating a poor QoL. GERD-HRQL is better at reflecting the symptoms than the generic questionnaire [36-Item Short Form Survey (SF-36)] [22,23]. The investigators translated the questionnaire into the Arabic language, tested it in a pilot study, and revised it to clarify any ambiguity. Information about age, gender, income, body mass index (BMI), smoking status, asthma, diabetes mellitus (DM), hypertension (HTN), hyperlipidemia, and any other related health disorders were also collected from each participant and further confirmed from the electronic health records. We categorized the participants into either poor QoL or good QoL based on their GERD-HRQL score. In terms of age, we grouped participants as follows - group A: 18-34 years; group B: 35-54 years; and group C: >54 years. The Institutional Review Board of the College of Medicine, King Saud University approved this study (Research project no. E-19-4417) on December 8, 2019. All the participants received a clear explanation about the study and all of them signed the informed consent form.

## Results

The total number of participants who completed the questionnaire was 200, indicating a response rate of 96.2%, and 58.5% (n=117) were males. Regarding the age groups, 43.5% (n=87) of participants fell in group B (35-54 years), 31.5% (n=63) in group A (18-34 years), and 25% (n=50) fell in group C (>54 years). As for monthly family income, about one-third of respondents (33.5%) earned 5,000-10,000 Saudi riyals. Smokers accounted for 21.5% of the participants, while 28% of participants were obese. Concerning the link between gender and the QoL in GERD patients, 46.2% of males had poor QoL, compared to 51.8% of females. The difference in QoL between genders was not statistically significant (p: 0.431).

Around 34.9% of participants in group A were found to have a poor QoL, compared to 57.5% of participants in group B, while 50% of participants in group C (>54 years) had a poor QoL, which was less than that in group B. There was a statistically significant difference in Qol between groups A and B (p: 0.006).

The study further categorized participants into four groups based on family income in Saudi riyals: below 5,000, between 5,000-10,000, between 11,000-15,000, and above 15,000. Around 44% of participants who had a family income below 5,000 riyals had poor QoL; 48.1% of participants who had a family income between 5,000-10,000 riyal had poor QoL, while 44.7% of participants who had a family income between 11,000-15,000 suffered from poor QoL; and 47.7% of participants who had a family income above 15,000 were found to have a poor QoL. The relationship between income and QoL in GERD patients was not statistically significant (p: 0.978) (Table 1).

**Table 1:** Comparison of health-related quality of life with respect to sociodemographic characteristics of study subjects (age, gender, and monthly income) *The difference between groups A and B GERD-HRQL: gastroesophageal reflux disease health-related quality of life

Variables		GERD-HRQL		
		Poor quality of life	Good quality of life	P-value
		N (%)	N (%)	
Gender	Male	54 (46.20%)	63 (53.80%)	0.431
	Female	43 (51.80%)	40 (48.20%)	
	Total	97 (48.50%)	103 (51.50%)	
Age groups* (years)	A (18-34)	22 (34.90%)	41 (65.10%)	0.006*
	B (35-54)	50 (57.50%)	37 (42.50%)	
	C (>54)	25 (50.00%)	25 (50.00%)	
	Total	97 (48.50%)	103 (51.50%)	
Monthly family income (Saudi riyal)	Below 5,000	11 (44.00%)	14 (56.00%)	0.978
	5,000-10,000	26 (48.10%)	28 (51.90%)	
	11,000-15,000	17 (44.70%)	21 (55.30%)	
	Above 15,000	21 (47.70%)	23 (52.30%)	
	Total	75 (46.60%)	86 (53.40%)	

The GERD-HRQL scores ranged from 2 to 46, with a mean value of 17.15 and a standard deviation (SD) of 9. The median score was 16. Hence, we considered participants with a score of 16 and above as having "poor QoL," while those with a score of less than 16 were determined to have "good QoL." About 39.5% of participants who were currently smoking had a poor QoL. The relationship between smoking and reduced QoL in GERD patients was not statistically significant (p: 0.184).

We classified the participants into three groups based on their BMI; normal-weight, overweight, and obese. About 34.7% of normal-weight participants had poor QoL, while 44.9% of overweight participants were found to have a poor QoL. Furthermore, 58.9% of obese participants had poor QoL; there was a statistically significant difference between Qol in normal-weight and obese groups (p: 0.013). In terms of diet, nearly 55.3% of participants on a high-fat diet had a poor QoL. The relation between a high-fat diet and reduced QoL in GERD patients was not statistically significant (p: 0.372).

The concomitant diseases that were studied were DM, HTN, dyslipidemia, and asthma. Approximately 50.9% of participants with DM had a poor QoL, while 56.9% of participants with HTN had a poor QoL. Also, 55.3% of participants with dyslipidemia had a poor QoL, and 51.1% of participants with asthma had a poor QoL. There was no significant relationship between the studied comorbidities and reduced QoL in GERD patients. (p: 0.67, 0.17, 0.35, and 0.69, respectively) (Table 2).

**Table 2:** The association of gastroesophageal reflux disease health-related quality of life with smoking, high-fat diet, body mass index, and other concomitant diseases *The difference between normal-weight and obese groups GERD-HRQL: gastroesophageal reflux disease health-related quality of life

Variables			GERD-HRQL		
			Poor quality of life, n (%)	Good quality of life, n (%)	P-value
Smoking	No		80 (51.00%)	77 (49.00%)	0.184
	Yes		17 (39.50%)	26 (60.50%)	
	Total		97 (48.50%)	103 (51.50%)	
High-fat diet	No		76 (46.90%)	86 (53.10%)	0.372
	Yes		21 (55.30%)	17 (44.70%)	
	Total		97 (48.50%)	103 (51.50%)	
Nutritional status	Normal weight		17 (34.70%)	32 (65.30%)	0.013*
	Overweight		22 (44.90%)	27 (55.10%)	
	Obese		33 (58.90%)	23 (41.10%)	
	Total		72 (46.80%)	82 (53.20%)	
Other concomitant diseases	Diabetes	No	68 (47.60%)	75 (52.40%)	0.671
		Yes	29 (50.90%)	28 (49.10%)	
		Total	97 (48.50%)	103 (51.50%)	
	Hypertension	No	68 (45.60%)	81 (54.40%)	0.166
		Yes	29 (56.90%)	22 (43.10%)	
		Total	97 (48.50%)	103 (51.50%)	
	Dyslipidemia	No	76 (46.90%)	86 (53.10%)	0.354
		Yes	21 (55.30%)	17 (44.70%)	
		Total	97 (48.50%)	103 (51.50%)	
	Asthma	No	73 (47.70%)	80 (52.30%)	0.688
		Yes	24 (51.10%)	23 (48.90%)	
		Total	97 (48.50%)	103 (51.50%)	

Based on the GERD-HRQL instrument that we used, the most frequent symptom affecting the QoL was "heartburn after meals" with a mean score of 2.56. On the opposite side of the spectrum, the least frequent symptom reported was "pain with swallowing" with a mean score of 0.61 (Table 3).

**Table 3:** Gastroesophageal reflux disease health-related quality of life questionnaire

Questions	No symptoms	Symptoms noticeable, but not bothersome	Symptoms noticeable and bothersome, but not every day	Symptoms bothersome every day	Symptoms affect daily activities	Symptoms are incapacitating, unable to do daily activities
	N (%)	N (%)	N (%)	N (%)	N (%)	N (%)
How bad is your heartburn?	10 (5.0%)	29 (14.5%)	73 (36.5%)	54 (27.0%)	20 (10.0%)	14 (7.0%)
Heartburn when lying down?	39 (19.5%)	27 (13.5%)	49 (24.5%)	51 (25.5%)	25 (12.5%)	9 (4.5%)
Heartburn when standing up?	66 (33.0%)	38 (19.0%)	57 (28.5%)	22 (11.0%)	14 (7.0%)	3 (1.5%)
Heartburn after meals?	17 (8.5%)	25 (12.5%)	56 (28.0%)	51 (25.5%)	34 (17.0%)	17 (8.5%)
Does heartburn change your diet?	66 (33.0%)	24 (12.0%)	28 (14.0%)	36 (18.0%)	26 (13.0%)	20 (10.0%)
Does heartburn wake you from sleep?	59 (29.5%)	29 (14.5%)	39 (19.5%)	36 (18.0%)	13 (6.5%)	24 (12.0%)
Do you have difficulty swallowing?	134 (67.0%)	21 (10.5%)	23 (11.5%)	7 (3.5%)	10 (5.0%)	5 (2.5%)
Do you have pain with swallowing?	142 (71.0%)	25 (12.5%)	17 (8.5%)	4 (2.0%)	9 (4.5%)	3 (1.5%)
Do you have bloating or gassy feelings?	126 (63.0%)	20 (10.0%)	19 (9.5%)	19 (9.5%)	9 (4.5%)	7 (3.5%)

## Discussion

According to the GERD-HRQL scale, 97 of GERD patients (48.5%) had poor QoL, while 103 participants (51.5%) had good QoL. The relationship between QoL and sociodemographic factors showed that QoL declines with age.

In terms of age, there was a statistically significant difference between group B and group A. This finding is comparable to a recent Irish survey on QoL [24]. As for gender, our study showed no significant difference between males and females in terms of QoL, which is consistent with a recent local study from Mecca [25]. On the contrary, an Irish report showed that females were more affected than males [24]. Regarding income, the difference in monthly income did not show any significant relationship with GERD patients' QoL; this finding is consistent with a previous study that showed no significant relationship in terms of income [13].

As expected, BMI had a significant effect on QoL, which is consistent with a recent polish study, which reported high BMI having a negative impact on QoL [26]. Our study did not show a significant relationship between a high-fat diet and QoL. However, other studies have reported a positive correlation between high fat intake and GERD symptoms [27,28].

Regarding smoking, unexpectedly, tobacco consumption did not show a significant effect on the QOL; interestingly, a recent study conducted in Mecca has shown similar findings [25]. Regarding other concomitant diseases such as DM, HTN, dyslipidemia, and asthma, none of these comorbidities showed a significant effect on the QOL, which appeared quite surprising; however, similar findings have been reported in the Mecca study [25].

We believe that monitoring the most important variables affecting the QoL of GERD patients may help in increasing awareness in the community regarding the same. Also, it may direct the healthcare system to tackle them, as part of Saudi Vision 2030, to achieve a vibrant society. Further research is necessary to find the most efficient ways to tackle these variables.

Our study has several limitations. Most importantly, as this study was cross-sectional, it could not determine causality. Another limitation was that the GERD-HRQL tool is not appropriate for measuring the atypical symptoms of GERD; specifically, there are no items in the questionnaire for respiratory or laryngeal symptoms, and for chest pain as a symptom independent of heartburn [23].

## Conclusions

We found that the QoL of patients with GERD was affected negatively by increasing age and high BMI. None of the other demographic variables and concomitant diseases had any significant effect on the QoL of the participants. Furthermore, we believe a health-strategy plan is needed to tackle obesity to improve the QoL of GERD patients.

## References

[1] Wiklund IK, Junghard O, Grace E (1998). Quality of Life in Reflux and Dyspepsia patients. Psychometric documentation of a new disease-specific questionnaire (QOLRAD). Eur J Surg Suppl.

[2] Al-Humayed SM, Mohamed-Elbagir AK, Al-Wabel AA, Argobi YA (2010). The changing pattern of upper gastro-intestinal lesions in southern Saudi Arabia: an endoscopic study. Saudi J Gastroenterol.

[3] Almadi MA, Almousa MA, Althwainy AF (2014). Prevalence of symptoms of gastroesophageal reflux in a cohort of Saudi Arabians: a study of 1265 subjects. Saudi J Gastroenterol.

[4] Alsuwat OB, Alzahrani AA, Alzhrani MA, Alkhathami AM, Mahfouz MEM (2018). Prevalence of gastroesophageal reflux disease in Saudi Arabia. J Clin Med Res.

[5] El-Serag HB, Sweet S, Winchester CC, Dent J (2014). Update on the epidemiology of gastro-oesophageal reflux disease: a systematic review. Gut.

[6] El-Serag HB (2007). Time trends of gastroesophageal reflux disease: a systematic review. Clin Gastroenterol Hepatol.

[7] (2020). World Gastroenterology Organisation (WGO): The WGO Foundation (WGO-F) - WGO Handbook on HEARTBURN: a global perspective. https://www.worldgastroenterology.org/UserFiles/file/WDHD-2015-handbook-final.pdf.

[8] Vakil N, van Zanten SV, Kahrilas P, Dent J, Jones R; Global Consensus Group (2006). The Montreal definition and classification of gastroesophageal reflux disease: a global evidence-based consensus. Am J Gastroenterol.

[9] Ohayon M, Milesi C, van Dijk M (2017). Comorbidity of chronic gastro-esophageal reflux (GERD) with sleep and medical disorders in a longitudinal survey of the American general population. Neurology.

[10] Farup C, Kleinman L, Sloan S, Ganoczy D, Chee E, Lee C, Revicki D (2001). The impact of nocturnal symptoms associated with gastroesophageal reflux disease on health-related quality of life. Arch Intern Med.

[11] Gisbert JP, Cooper A, Karagiannis D, Hatlebakk J, Agréus L, Jablonowski H, Zapardiel J (2009). Impact of gastroesophageal reflux disease on patients’ daily lives: a European observational study in the primary care setting. Health Qual Life Outcomes.

[12] Singh S, Garg SK, Singh PP, Iyer PG, El-Serag HB (2014). Acid-suppressive medications and risk of oesophageal adenocarcinoma in patients with Barrett's oesophagus: a systematic review and meta-analysis. Gut.

[13] Maleki I, Masoudzadeh A, Khalilian A, Daheshpour E (2013). Quality of life in patients with gastroesophageal reflux disease in an Iranian population. Gastroenterol Hepatol Bed Bench.

[14] Jarosz M, Taraszewska A (2014). Risk factors for gastroesophageal reflux disease: the role of diet. Prz Gastroenterol.

[15] Mohammed I, Cherkas LF, Riley SA, Spector TD, Trudgill NJ (2003). Genetic influences in gastro-oesophageal reflux disease: a twin study. Gut.

[16] Cameron AJ, Lagergren J, Henriksson C, Nyren O, Locke GR 3rd, Pedersen NL (2002). Gastroesophageal reflux disease in monozygotic and dizygotic twins. Gastroenterology.

[17] (2020). Quality of life: everyone wants it, but what is it?. https://www.forbes.com/sites/iese/2013/09/04/quality-of-life-everyone-wants-it-but-what-is-it/.

[18] Karimi M, Brazier J (2016). Health-related quality of life, and quality of life: what is the difference?. Pharmacoeconomics.

[19] Altwigry AM, Almutairi MS, Ahmed M (2017). Gastroesophageal reflux disease prevalence among school teachers of Saudi Arabia and its impact on their daily life activities. Int J Health Sci (Qassim).

[20] Affairs C of E and D (2020). Saudi Vision 2030. Saudi Vision 2030.

[21] (2020). Saudi Vision 2030: quality of life program 2020 - delivery plan. https://vision2030.gov.sa/sites/default/files/attachments/QoL%20English_0.pdf.

[22] Velanovich V (1998). Comparison of generic (SF-36) vs. disease-specific (GERD-HRQL) quality-of-life scales for gastroesophageal reflux disease. J Gastrointest Surg.

[23] Velanovich V (2007). The development of the GERD-HRQL symptom severity instrument. Dis Esophagus.

[24] Quigley EM, Hungin AP (2005). Review article: quality-of-life issues in gastro-oesophageal reflux disease. Aliment Pharmacol Ther.

[25] Algethami SS, Alosaimi HS, Al-Malki MA (2018). Gastroesophageal reflux disease among pilgrims during the Hajj period (1438 Hegira): prevalence and impact on the quality of life. Egypt J Hosp Med.

[26] Gorczyca R, Pardak P, Pękala A, Filip R (2019). Impact of gastroesophageal reflux disease on the quality of life of Polish patients. World J Clin Cases.

[27] Oliveria SA, Christos PJ, Talley NJ, Dannenberg AJ (1999). Heartburn risk factors, knowledge, and prevention strategies: a population-based survey of individuals with heartburn. Arch Intern Med.

[28] Bolin TD, Korman MG, Hansky J, Stanton R (2000). Heartburn: community perceptions. J Gastroenterol Hepatol.

